# Measurement of bunch length and temporal distribution using accelerating radio frequency cavity in low-emittance injector

**DOI:** 10.1038/s41598-020-76054-w

**Published:** 2020-11-03

**Authors:** Ji-Gwang Hwang, Tsukasa Miyajima, Yosuke Honda, Eun-San Kim

**Affiliations:** 1 Helmholtz-Zentrum Berlin (HZB), Albert-Einstein straße 15, 12489 Berlin, Germany; 2 KEK, High Energy Accelerator Research Organization, 1-1 Oho, Tsukuba, Ibaraki, 305-0801 Japan; 3 Department of Accelerator Science, Korea University Sejong Campus, Sejong, 339-700 South Korea

**Keywords:** Plasma physics, Statistical physics, thermodynamics and nonlinear dynamics, Techniques and instrumentation

## Abstract

We demonstrate an experimental methodology for measuring the temporal distribution of pico-second level electron bunch with low energy using radial electric and azimuthal magnetic fields of an accelerating ($\hbox {TM}_{01}$ mode) radio frequency (RF) cavity that is used for accelerating electron beams in a linear accelerator. In this new technique, an accelerating RF cavity provides a phase-dependent transverse kick to the electrons, resulting in the linear coupling of the trajectory angle with the longitudinal position inside the bunch. This method does not require additional devices on the beamline since it uses an existing accelerating cavity for the projection of the temporal distribution to the transverse direction. We present the theoretical basis of the proposed method and validate it experimentally in the compact-energy recovery linac accelerator at KEK. Measurements were demonstrated using a 2-cell superconducting booster cavity with a peak on-axis accelerating field ($E_0$) of 7.21 MV/m.

## Introduction

Future accelerators, such as X-ray free-electron lasers (FEL)^1–7^, energy recovery linacs (ERL)^8–11^, and linear colliders^12–15^, are aiming at high-brightness and high-brilliance. This can be accomplished by a high-performance injector that can produce electron beams with an ultra-low emittance in 6-D phase space. This scientific motivation stimulates the development of high-power and low-emittance photo-injectors^16–23^ that consist of a superconducting linear accelerator capable of producing a continuous stream of electron bunches at repetition rates of a few MHz. These injectors also open new horizons for many applications in the physical sciences, materials science, chemistry, health, information technology and security^24–28^. For these injectors, measurements of the temporal profiles of electron beams are important for achieving the desired performance. Many instruments and technologies, such as streak cameras^29–31^, transverse deflecting cavities^32–36^, electro-optic probes^37,38^, Terahertz streaking^39,40^ and devices based on the coherent property of synchrotron radiation^41–48^, have been devised for measuring and monitoring the longitudinal parameters of picosecond (ps) and sub-picosecond electron bunches, have been applied in accelerators. These methods, however, require construction space for installing complex and expensive equipment, such as sophisticated streaking devices with highly accurate timing systems, high-quality optical elements, or deflecting cavities with high power sources.

We propose a novel technique for measuring few-ps level bunch lengths by using radial electric and azimuthal magnetic fields of an accelerating ($\hbox {TM}_{01}$ mode) RF cavity which is well known for the source of second-order ponderomotive focusing force^49^. The advantage of this method is that it only requires a corrector magnet installed upstream in an accelerating RF cavity for manipulating a beam offset inside the cavity, and a profile monitor installed downstream in the cavity for measuring a transverse beam distribution. Therefore, injectors of accelerators worldwide, which have low energy beams with an accelerating RF cavity, can use this method for measuring the longitudinal distribution as well as few-ps bunch lengths without installing any specialized devices. We present the theoretical basis and estimation of the resolution for our method. In addition, the projection of the longitudinal distribution (which is a semi flat-top distribution with 13.51 ps (rms)) to the transverse direction has been demonstrated at the cERL^50^ which has 2-cell superconducting radio frequency (SRF) cavities in an injector. The result of the measurement for a bunch length of 3.3 ps (rms), which is closest to the resolution of the system, is also verified experimentally.

## Methods

**Figure 1:**
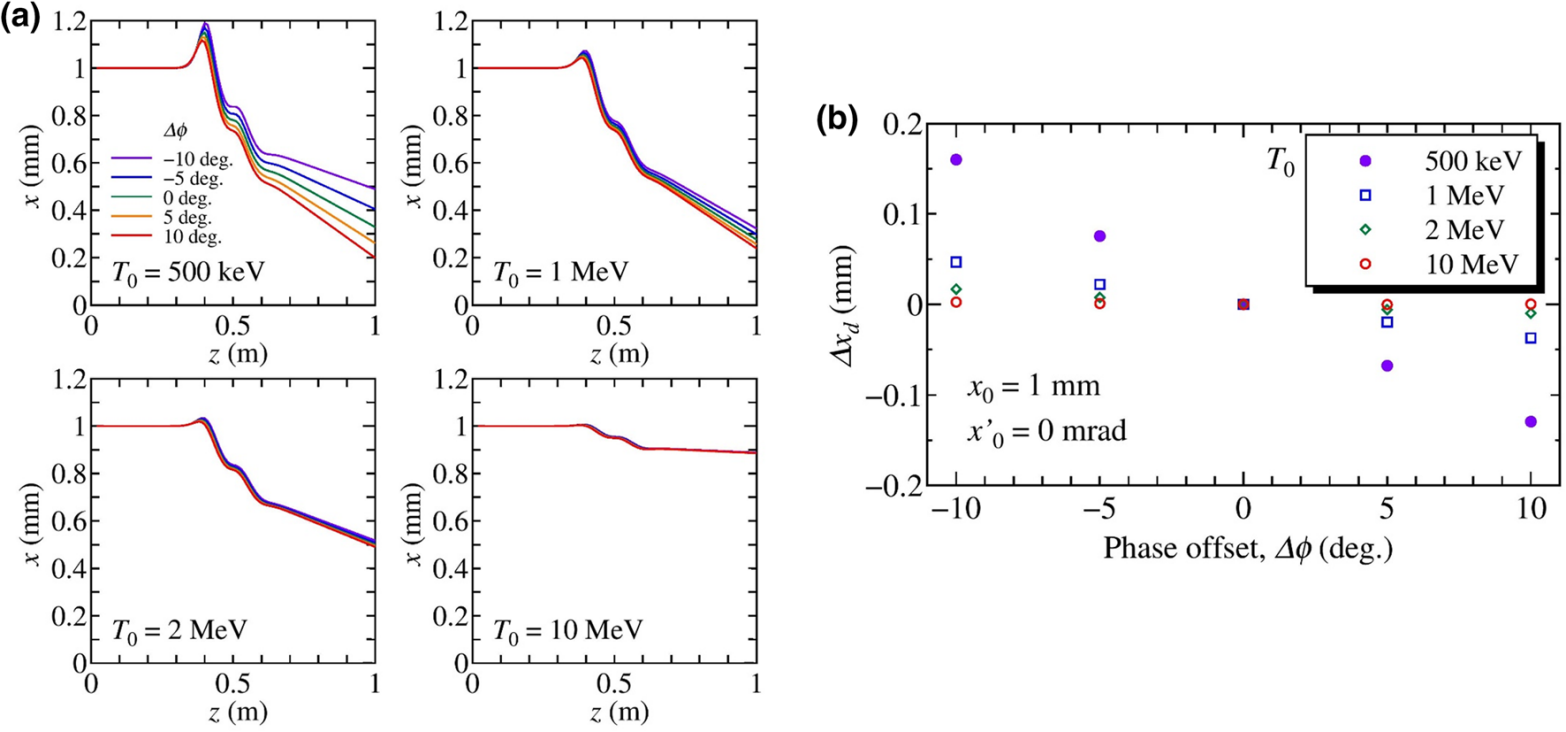
(**a**) Beam trajectories as a function of $\Delta \phi = \omega \Delta t$ with the initial condition of $x_0 = 1$ mm and $x'_0 = 0$ mrad. The initial kinetic energies are $T_0$ = 500 keV, 1 MeV, 2 MeV and 10 MeV, respectively. (**b**) Relative deflected positions at the end of the beamline as a function of $\Delta \phi$. The relative position is calculated from the position for the on-crest phase, $\Delta \phi = 0$.

Our proposed method uses the time-dependent radial electric and azimuthal magnetic fields component of the $\hbox {TM}_{01}$ mode of an accelerating cavity to project the temporal distribution to a transverse direction. The accelerating $\hbox {TM}_{01}$ mode only has longitudinal fields at the on-axis so they cannot be used to deflect the beam when electron bunches passes the center of an accelerating cavity. However, the radial electric and azimuthal magnetic fields, which can provide a phase-dependent transverse kick to electron beams, arise at the off-axis of the cavity^51^. Using the paraxial approximation, the equation of motion with the maximum acceleration phase (on-crest phase) is given by

1 $$\frac{dp_r}{dt} = e(E_r-\beta c B_\theta ) = -\frac{er}{2} \left[ \frac{\partial E_m(z)}{\partial z} \sin (\omega (t + \Delta t) - \phi _m(T_0)) +\frac{\beta \omega }{c} E_m(z) \cos (\omega (t + \Delta t) + \phi _m(T_0)) \right] ,$$

where $\beta = v/c$, and $E_r$ and $B_\theta$ are the radial electric field and azimuthal magnetic fields, respectively, $E_z = E_m(z) \sin (\omega t + \phi _0)$, $\omega = 2 \pi f$ where *f* is the cavity frequency, $\phi _0$ is a phase offset that adjusts the acceleration phase, $\phi _m(T_0)$ is the on-crest phase at the initial kinetic energy $T_0$, *r* is the beam offset, and $\Delta t$ is the time difference from $t = 0$ at the entrance of the beamline. Equation (1) indicates that the transverse force is a linear function of $r$ around the $z$-axis. Therefore, we can describe the transverse motion using the transfer-matrix formalism, which is given by $x_1 = M x_0$. Here $x_0 \equiv (x_0, x'_0)^T$ and ${x}_1 \equiv (x_1, x'_1)^T$ are the initial and final coordinates in the horizontal phase-space at the entrance and exit of the beamline, respectively, $x' \equiv dx/dz$, and $M$ is a 2$\times$2 transfer matrix. The equation of motion for the vertical axis is equivalent since the cavity is cylindrically symmetric. For high energy beams $\left( \beta \simeq 1\right)$, the transfer matrix can be calculated analytically^49^. In contrast, the transverse motion of low energy beams is complicated by the significant changes in the velocity of an electron inside the cavity owing to its low initial kinetic energy and then the trajectories have been numerically simulated using General Particle Tracer (GPT) code^52^ with the electromagnetic field of the 2-cell SRF cavities calculated by using the Poisson/Superfish code^53^ (Figures S1 and S2 in the Supplementary Appendix). The electron with $\Delta t = 0$ is accelerated with the on-crest phase. and then the time difference corresponds to the RF phase difference from the on-crest phase, $\Delta \phi = \omega \Delta t$. In this work, degrees are used as the unit of the RF phase and the angular frequency can be represented as $\omega = 360 \times f$ in the units of deg./s. A particle tracking simulation was performed for two initial conditions with $x_0 = 1$ mm and $x'_0$ = 0 mrad at different energies $T_0$ = 500 keV, 1 MeV, 2 MeV and 10 MeV to evaluate the effect of the time difference on the transverse trajectory. The transverse trajectories and relative deflected positions as functions of the energy and the phase are shown in Fig. 1a,b, respectively.

**Figure 2:**
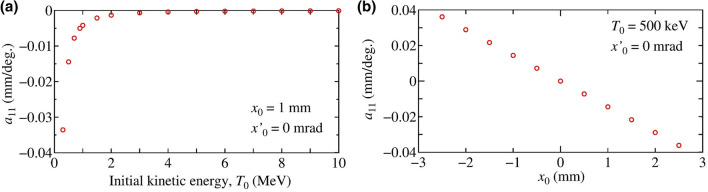
Deflection coefficient $a_{11}$ as functions of (**a**) the initial kinetic energy $T_0$ with initial conditions of $x_0 = 1$ mm and $x'_0 = 0$ mrad, and (**b**) the initial offset $x_0$ with an initial kinetic energy of 500 keV and an initial angle of $x'_0$ = 0 mrad.

In the case of a low-energy beam, whose $T_0$ = 500 keV, the final position $x_1$ depends on $\Delta \phi$. This is the origin of the deflection force which is utilized in the proposed method. The transverse forces experienced by the electron when $T_0$ = 500 keV and 10 MeV are shown in Fig. S3 (see the Supplementary Appendix). The $\Delta x_d(\Delta \phi )$ is a linear function of $\Delta \phi$ around the on-crest phase. In addition, for higher $T_0$, the position variation decreases significantly, indicating that the deflection effect is effective for low energy beams. Using on these results, the relative position displacement can be expressed as

2 $$\Delta x_d(\Delta \phi )= x_1(\Delta \phi ) - x_1(0) = a_{11} \Delta \phi ,$$

where $a_{11}$ is a linear coefficient of $\Delta x_d(\Delta \phi )$ which describes the strength of the deflection effect. In order to analyze effects of the initial kinetic energy $T_0$ and the initial offset $x_0$, the deflection coefficient was simulated as a function of the $T_0$ with an initial offset $x_0$ of 1 mm and $x_0$, with an initial kinetic energy of 500 keV, respectively. The dependence on the initial kinetic energy and offset is shown in Fig. 2.

**Figure 3:**
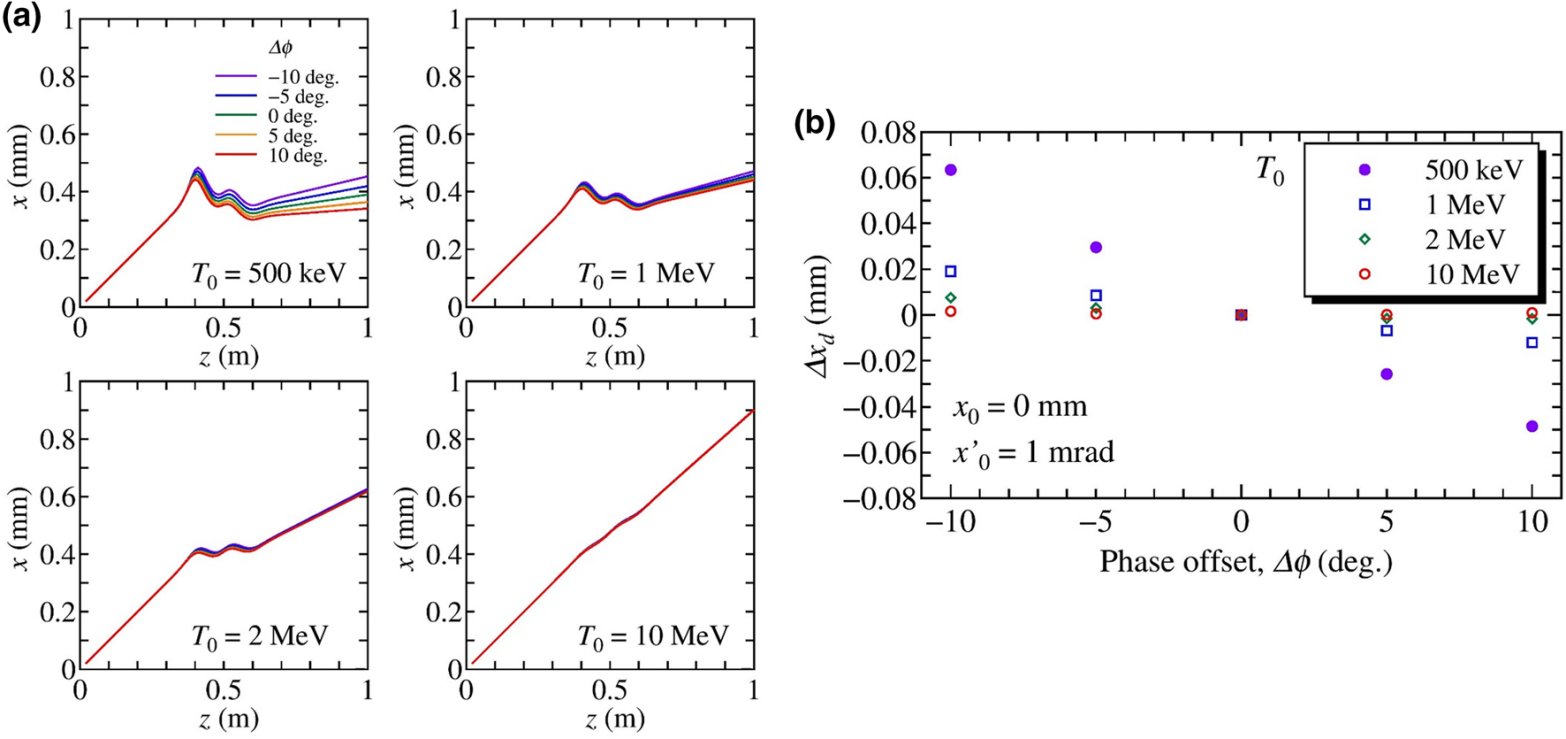
(**a**) Beam trajectories as a function of $\Delta \phi$ with the initial condition of $x_0 = 0$ mm and $x'_0 = 1$ mrad. The initial kinetic energies are $T_0$ = 500 keV, 1 MeV, 2 MeV and 10 MeV, respectively. (**b**) Relative deflected positions at the end of the beamline as a function of $\Delta \phi$. The relative position is calculated from the position for the on-crest phase, $\Delta \phi = 0$.

**Figure 4:**
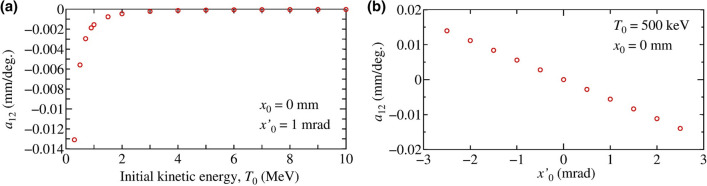
Deflection coefficient $a_{12}$ as functions of (**a**) the initial kinetic energy $T_0$ with the initial condition of $x_0 = 0$ mm and $x'_0 = 1$ mrad, and (**b**) the initial angle $x'_0$ with an initial kinetic energy of 500 keV and an initial offset of $x_0$ = 0 mm.

The simulation results indicate that the deflection coefficient $a_{11}$ depends on the initial offset since the amplitude of transverse electric-field inside the cavity is a linear function of the offset, according to Eq. (1) and the deflection effect is negligible for high energy beams. Therefore, in our proposed method, the deflection effect is effective when $T_0 < 2$ MeV. Hence, in terms of $x_0$ and $\Delta \phi$, the deflection effect becomes:

3 $$\Delta x_d(\Delta \phi ) = d_{11} x_0 \Delta \phi ,$$

where the $d_{11}$ is a coefficient associated with the initial offset. We analyze the effect of the initial angle, $x'_0$, since it introduces the beam offset while traveling through the cavity. Numerical simulations are performed using the initial condition $x_0 = 0$ mm and $x'_0$ = 1 mrad to deconvolute the effect of the initial offset. The transverse trajectories, corresponding to $T_0$ = 500 keV, 1 MeV, 2 MeV and 10 MeV, as a function of $\Delta \phi$ were simulated. The results of beam trajectories and relative deflected position at the end of the beamline as a function of $\Delta \phi$ are shown in Fig. 3a,b, respectively. For a low energy beam, the trajectory changes depending on the time difference from the on-crest phase, indicating that the initial angle also affects the deflection force. The $\Delta x_d(\Delta \phi )$ is a linear function of $\Delta \phi$ around $\Delta \phi = 0$. The relative positional displacement can be expressed as a function of $\Delta \phi$ as

4 $$\Delta x_d(\Delta \phi ) = a_{12} \Delta \phi ,$$

where $a_{12}$ is a coefficient associated with the initial angle. In order to analyze effects of the initial kinetic energy $T_0$ and the initial angle $x'_0$, the deflection coefficient was simulated as functions of the $T_0$ with an initial offset $x'_0$ of 1 mrad, and the $x'_0$ with an initial kinetic energy of 500 keV, respectively. The dependence on the initial kinetic energy shown in Fig. 4 indicates that the deflection effect associated with the initial angle is effective for $T_0 < 2$ MeV, similar to the effect associated with the initial position, $x_0$. Then, we can define $a_{12}(x'_0) = d_{12} x'_0$, where $d_{12}$ is a coefficient associated with the initial angle. Thus, the deflection effect associated with the initial angle becomes:

5 $$\Delta x_d(\Delta \phi ) = d_{12} x'_0 \Delta \phi .$$

### Deflection effect for low energy electron

Based on the results of the analysis of the deflection effect as functions of the initial position and initial angle presented in the previous section, we can summarize the deflection effect for low energy beams with small phase deviations as

6 $${ x}_1 = M { x}_0 + \Delta \phi D { x}_0,$$

Here, $M$ is a linear transfer matrix for the on-crest phase whose elements are $m_{11}$, $m_{12}$, $m_{21}$ and $m_{22}$, and $D$ is a 2 $\times$ 2 matrix with the elements $d_{11}$, $d_{12}$, $d_{21}$ and $d_{22}$. $D$ indicates the strength of the deflection effect which can be determined by numerical simulations or experimental measurements. The beam position at the exit of the beamline is described by

7 $$x_1(\Delta \phi ) = x_c + a_1 \Delta \phi = x_c + (d_{11} x_0 + d_{12} x'_0) \omega \Delta t.$$

where $x_c = m_{11} x_0 + m_{12} x'_0$. The strength of the deflection effect can be controlled by $x_0$ and $x'_0$. For bunch length measurements, the coefficient $a_1$ can be measured by scanning the phase offset, $\Delta \phi$. The temporal distribution of the electron bunch with respect to $\Delta t$ is projected on the transverse distribution. This only considered the deflection effect for a single electron with the time difference, $\Delta t$. Here, we consider an electron bunch with a temporal distribution $f(\tau )$ at the entrance of the beamline, and a horizontal distribution $g(x)$ at its exit. The horizontal distribution can be measured by a profile monitor located at the exit of the beamline. The parameter $\tau$, which is the time difference from the center of the electron bunch is adjusted to the maximum acceleration condition. The horizontal distribution, which can be measured using the profile monitor installed at the exit of the beam line, convoluted by the initial temporal distribution, is given by

8 $$G(x) = \int f(\tau ) g(x - (d_{11} x_0 + d_{12} x'_0) \omega \tau ) d\tau .$$

In order to calculate the projection of the bunch length to the transverse beam size, we assume that the electron beam in the longitudinal phase space follows a Gaussian distribution, and is transversally small compared to the transverse radius of the RF cavity. We describe the transverse beam size at the profile monitor as functions of the bunch length and accelerating RF cavity parameters as

9 $$\sigma _x = \sqrt{\sigma _{x0}^2 + ((d_{11} x_0 + d_{12} x'_0) \omega \sigma _t)^2},$$

where $\sigma _t$ is the rms bunch length, and $\sigma _{x0}$ is the rms beam size when the electron bunch passes the center of the accelerating RF cavity. As a consequence of Eq. (9), the transverse beam size at the profile monitor is proportional to the square root of the bunch length. In addition, Eq. (9) represents the essence of the measurement of the bunch length using the accelerating RF cavity. The deflection coefficient $a_1$ is a crucial parameter because it affects the correlation between the longitudinal and transverse directions. The parameters $x_0$ and $x'_0$ are also important for controlling the strength of the deflection effect since $a_1$ is a linear function of both parameters. In our method, the deflection coefficient is determined experimentally. The initial beam offset, $x_0$, and the angle, $x'_0$, are controlled in accordance with the resolution required for the bunch length.

### Resolution of bunch length measurement

The estimation of the temporal resolution is important^54^ for validating the proposed method. The temporal resolution, $R_t$, can be defined as the bunch length that yields on the profile monitor, a transverse beam size equal to the transverse resolution of the monitor itself. In this case, the transverse resolution for the bunch length measurement is $\sigma _{x0} + \sigma _{c}$, where $\sigma _{c}$ is the transverse resolution of the beam size measured by the profile monitor. This limits the resolution of the bunch length. According to Eq. (9) the temporal resolution of the proposed method is given by

10 $$R_t = \frac{1}{\left| d_{11} x_0 + d_{12} x'_0\right| \omega } \sqrt{\sigma _{c}^2 + 2 \sigma _{x0} \sigma _{c}}.$$

The temporal resolution of the proposed method depends on the deflection coefficient which, in turn, depends on the initial beam energy, amplitude of the electric field, and initial beam size, $R_{12}$, which is dominated by the distance and devices between the accelerating RF cavity and the profile monitor, and the beam offset inside the accelerating RF cavity. Among these parameters, the deflection coefficient and beam offset are important, because they can be controlled independently of the conditions of the beam transportation line. Since the deflection coefficient is determined by the design of the RF cavity, the beam offset inside it is an important parameter for controlling the temporal resolution of our method.

Since the method requires the beam offset inside the RF cavity, the kick effect owing to the transverse wakefield should be considered. The kick angle resulting from the transverse wakefield is obtained using^55–57^

11 $$\Delta r'(s) = \frac{e I_0 L r}{c E_k} \int _{0}^{s} ds'\ W_{tr}(s'),$$

where *L* is the length of the beam pipe, *r* is the beam offset from the center of the cavity, $E_k$ is the beam energy, $I_0$ is the beam current, and $W_{tr} = 4 Z_0 c s_0 \phi (s) /\pi a^4 \left[ 1- \left( 1+\sqrt{s/s_0}\right) \exp \left( -\sqrt{s/s_0} \right) \right]$. In addition, $Z_0$ = 120 $\pi \Omega$, *a* is the radius of the iris, $\phi (s)$ is the step function, $s_0=g/8\left( a/(\alpha (g/L)L)\right) ^2$, where *g* is the gap, and $\alpha (\gamma ) \sim 1- \alpha _1 \sqrt{\gamma }-(1-2\alpha _1)\gamma$ with $\alpha _1 = 0.4648$. Although the wakefield model is valid for ultra-relativistic beams ($\beta$ $\sim$ 1) with an elongated structure longer than the catch-up distance, it probably gives the most accurate estimation of the order of magnitude of the kick angle arising from the wakefield. In our case, the kick angle corresponding to a beam offset of 10 mm, bunch charge of 10 fC, iris radius of 3.37 cm, bunch length of 3 ps, and energy of 0.39 MeV is lower than 0.04 $\mu$rad, i.e., negligible. It was verified experimentally that the emittance growth owing to the radial electric-field is significantly greater than the contribution by long-range and short-range wakefield effects^58^.

**Figure 5:**
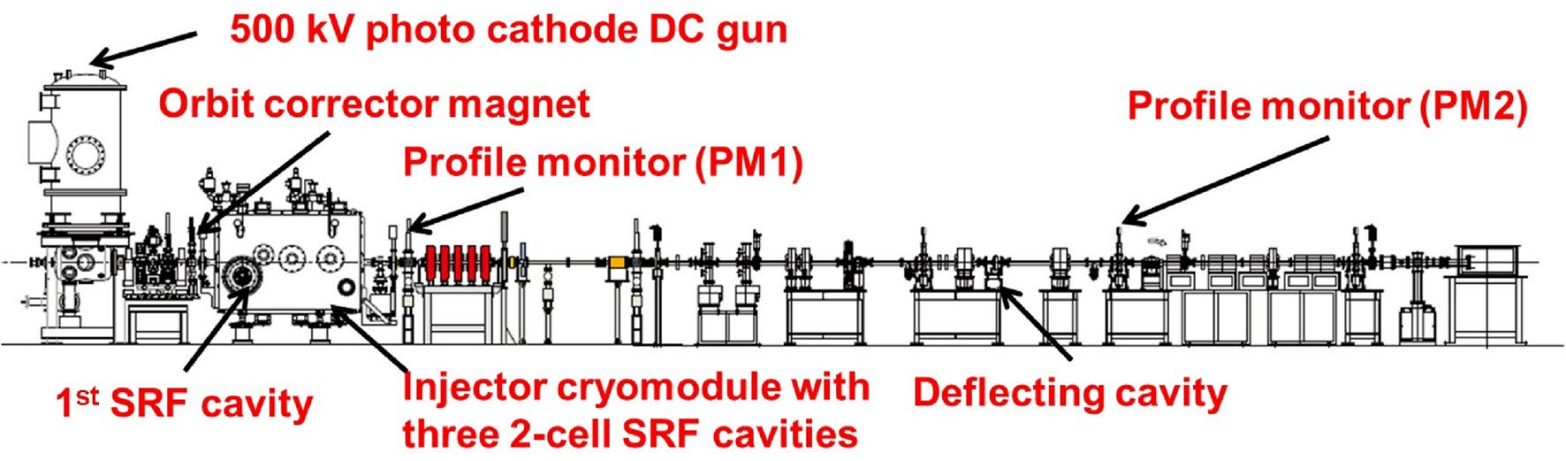
Layout of an injector beam line for the cERL.

### Experimental setup at cERL

**Figure 6:**
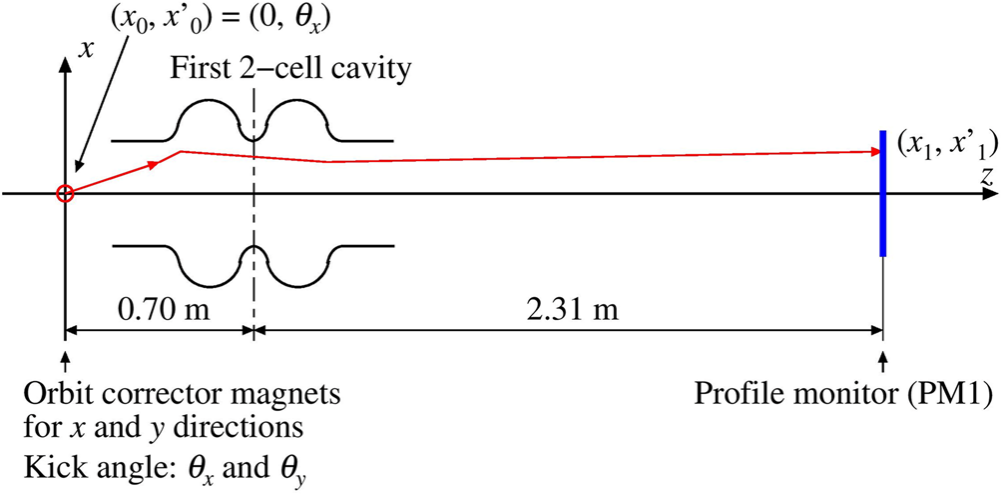
Schematic view of the bunch length measurement in the cERL injector. Corrector magnets for the horizontal and vertical directions, located at the entrance, control the kick angles $\theta _x$ and $\theta _y$. The transverse profile is measured by a profile monitor (PM1).

This technique is demonstrated in a high-performance cERL injector^59^, which consists of a 500 kV photo cathode DC gun^60–62^ and three 2-cell SRF cavities^63,64^. The cERL injector can produce electron bunches with a repetition rate of 1.3 GHz and bunch charges from a few fC to pC. Macro-pulses have burst lengths of 0.1-1.2 $\mu$s and repetition rate of 5 Hz^10^. The layout of the cERL injector is shown in Fig. 5. The deflection coefficient $a_{1}$ of the cavity can be determined experimentally by measuring the relation between the RF phase of the cavity and the displacement of the central position at a profile monitor $X_c$ installed downstream in the cavity. Using Eq. (4), the coefficient can be represented as $a_1 = \Delta x_d(\Delta \phi )/\Delta \phi = (d_{11} x_0+ d_{12} x'_0)$. Since we introduce the initial beam angle $x'_0$ by adjusting the strength of the corrector magnet installed upstream in the cavity during the experiment, the coefficient can be approximated to $a_1 = (d_{12} x'_0)$. The schematic layout for measuring the deflection coefficient is depicted in Fig. 6.

Since the deflection coefficient is linearly proportional to the initial beam angle $x'_0$, offset $x_0$, and effective accelerating field of the cavity, the beam and machine parameters are evaluated carefully. During calibration, the gun voltage was set to 390 kV and provide a 3.3 ps (rms) laser pulse on the GaAs cathode to produce a 10 fC charged electron bunch^65^. After tuning the gun component of the injector, the corrector magnet used to control the beam offset inside the RF cavity was calibrated using the profile monitor (PM1) installed in the downstream of the cavity as shown in Fig. 5. Next, the strength of the corrector magnet was set to the beam passing the accelerating RF cavity with a reasonable beam offset, 7.85 mm^58^, and the beam position was measured at the profile monitor while the phase of the accelerating RF cavity was changed.

**Figure 7:**
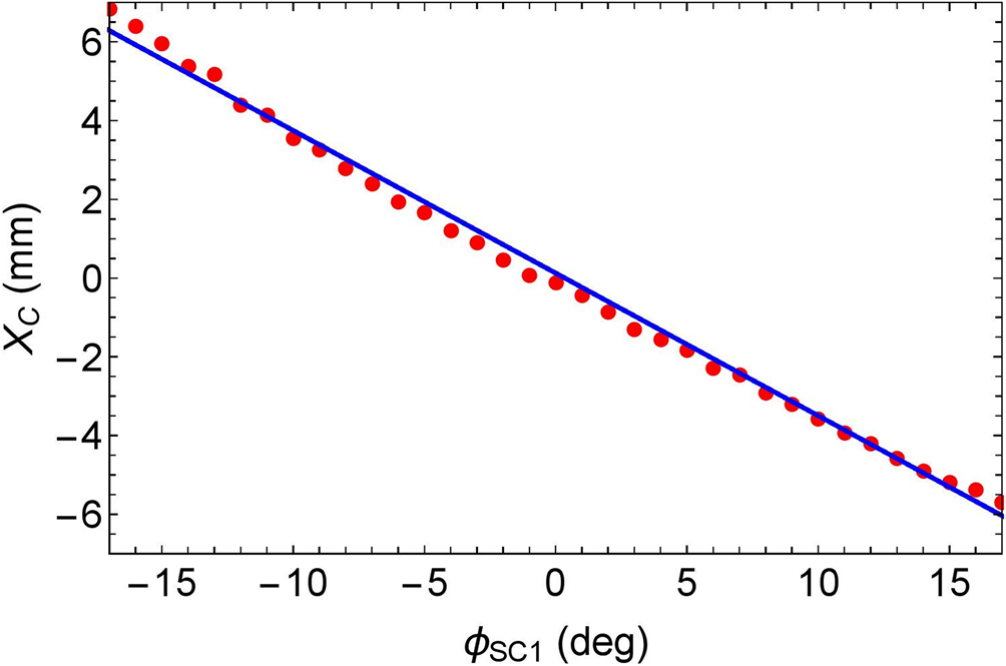
Result of the measurement of the displacement of the beam position at the profile monitor as a function of the RF phase.

Figure 7 shows the result of the measurement of the displacement as a function of the RF phase. These measurements were used to calibrate the coefficient $d_{12} = a_{1}/x'_0$, yielding a value of $-0.0324 \pm 0.00026$ mm/deg/mrad. Under this condition, the beam is accelerated from 390 keV to 1.86 MeV.

A reasonable beam offset that matces the resolution required for the bunch length measurement by our system can be estimated based on the calibrated deflection coefficient. In our system, the resolution of the transverse beam size measurement using the profile monitor $\sigma _c$ is smaller than 0.10 mm, and the initial beam size when the electron beam passes the center of the accelerating RF cavity, $\sigma _{x0}$, is 0.469 mm. These these parameters were used to calculate the resolution as a function of the beam offset based on Eq. (10). In order to measure the bunch length of 3.3 ps (rms), the beam offset inside the accelerating RF cavity should be larger than 4.80 mm. The energy variation by the phase shift due to the beam offset is negligible. We note that the beam offset should be kept as small as possible during the experiment to avoid the effects of the transverse wakefield and non-linearity for a large offset even though it controls the resolution significantly. In our case, the maximum beam offset is limited by the inner radius of the RF cavity, which is about 35 mm. Assuming that the initial beam angle is 16 mrad, corresponding to a beam offset equivalent to 1/3 of the cavity radius, the resolution of the proposed method is estimated to be about 0.98 ps using Eq. (10). The resolution can be improved by increasing the value of $d_{12}$ coefficient which can be achieved by increasing the field gradient of the cavity or lowering the initial beam energy.

## Results

We demonstrated the projection of the longitudinal profile to the transverse direction by an accelerating RF cavity with a beam offset inside it by measuring of the temporal profile with a long bunch, in which eight Gaussian pulses with a bunch length of 3.3 ps (rms) were stacked. In the experiment, the first cavity was turned on and the peak on-axis accelerating field of the accelerating RF cavity, $E_0$, was set to 7.21 MV/m. The second and third cavities were turned off, and the beam corrector magnet, installed upstream in the cavity, was adjusted to produce horizontal or vertical beam offsets of 16 mm from the electromagnetic center of the cavity. The beamline is a non-dispersive section. Since the electron beam produced by the DC gun has a energy spread of lower than 0.01 %, the dispersion caused by the corrector magnet in the beamline, which leads to variations in the beam size, can also be ignored. The electromagnetic center of the cavity was estimated by measuring the $dX_C/d\phi$ and $dY_C/d\phi$ values that were derived in Ref.^58^.

**Figure 8:**
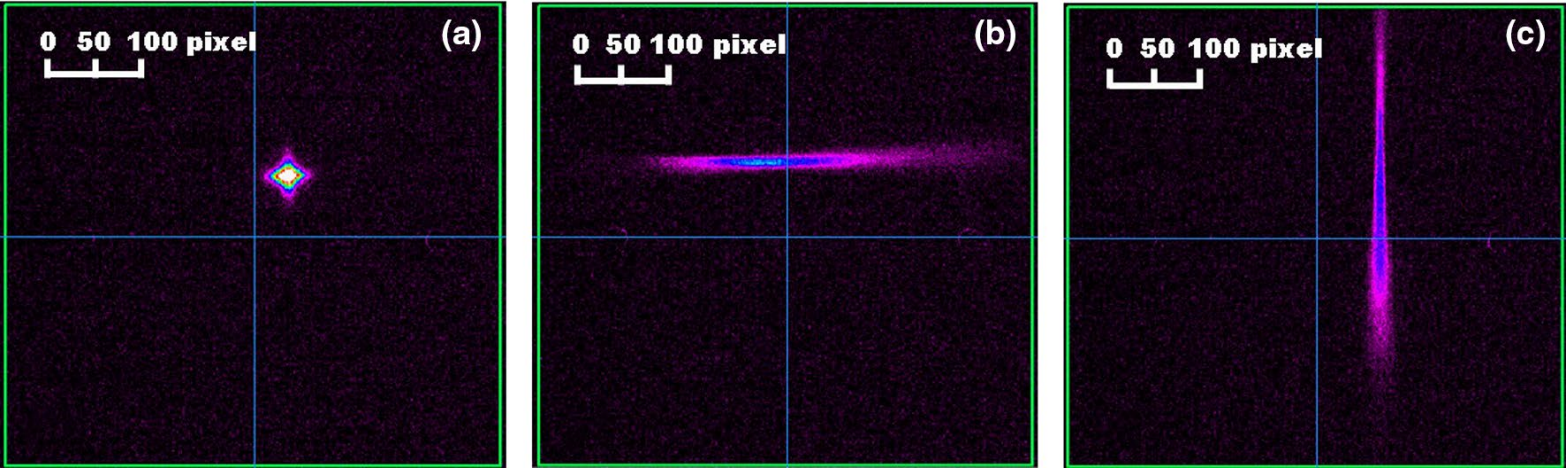
Measured transverse beam profiles with a bunch length of 13.51 ps (rms) for (**a**) zero offset, (**b**) non-zero horizontal beam offset, $\Delta x = 16.1$ mm, and (**c**) non-zero vertical beam offset, $\Delta y = 16.0$ mm in the cavity. Each pixel on the profile monitor corresponds to 54.9 $\mu$m.

**Figure 9:**
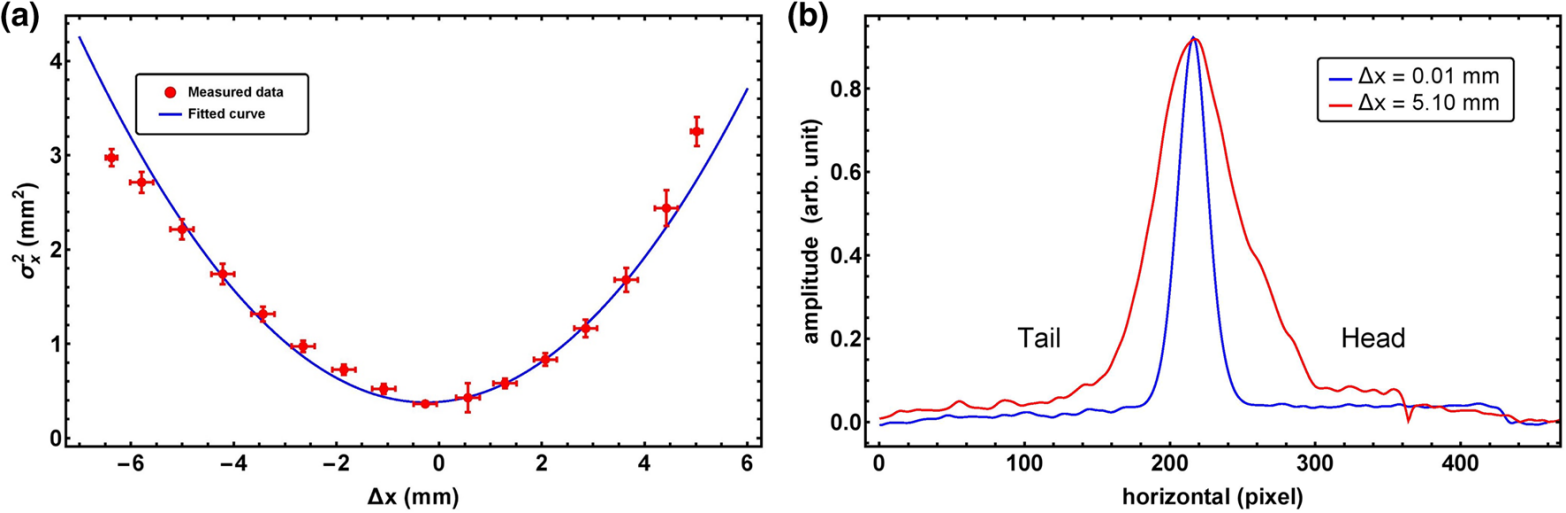
Result of the measurement of (**a**) the relation between the horizontal beam offset and the beam size at the profile monitor for a long bunch and (**b**) temporal profile with beam offsets of 0.01 mm and 5.1 mm.

The top graph of Fig. 8 shows the transverse profile measured by the profile monitor (PM1) for zero offset inside the cavity. In this case, the beam passed through the electromagnetic center of the cavity, and the measured profile was almost same as the original transverse beam size. This shows a lack of correlation between the longitudinal and transverse directions owing to the deflecting force. This also indicates that the electromagnetic center of the cavity was accurately estimated by measuring $dX_C/d\phi$ and $dY_C/d\phi$^58^. The lower graphs of Fig. 8 show the transverse profiles with horizontal or vertical offsets of 16 mm from the center. In these cases, the longitudinal distribution was projected to the horizontal or vertical direction. These results show that the non-zero beam offset inside the cavity causes the correlation between the longitudinal and transverse directions, thereby validating the principle of our method. Moreover, the variation of the beam size variation as a function of the beam offset inside the cavity was measured to estimate the length of the temporal profile. The result is shown in Fig. 9. The minimum beam size corresponds to the zero offset case.

In order to analyze the measured data, Eq. (9) can be expressed as

12 $$\sigma _x^2 = \sigma _{x0}^2 + \xi (\Delta x + \chi )^2,$$

where $L_0$ is the distance between the corrector magnet and the center of the cavity, $\Delta x$ is the beam offset at the center of the cavity, and $\chi$ is a constant that represents the minimum beam size position. Since this method measures a relative beam size change respect to $\sigma _0$ in Eq. (12) which is determined by the integration of complex physics processes including optics, energy variation as well as low-energy beam dynamics, it is no longer necessary to deconvolute the emittance dilution by space charge effects. The $\xi$ can be represented as $\xi = (d_{12}\omega \sigma _z/L_0 )^2$ because the beam is aligned to the center of cavity $x_0 =0$. Using the fitted curve plotted in Fig. 9, which is derived from Eq. (12), the $\xi$ value was calculated to be $0.0850 \pm 0.000348$. The beamline parameters involves the conversion of the variation of the beam size as a function of the beam offset inside the cavity to the rms bunch length. In this experiment, the $E_p$, $E_c$, $R_{12}$, and $\Delta \phi$ were 1.86 MeV, 0.39 MeV, 2.308 m, and 0$^{\circ }$, respectively. The parameters $E_c$ and $E_p$ are the beam energies at the entrance of the RF cavity and profile monitor, respectively. Using these parameters, our method yielded a bunch length of $13.51 \pm 0.03$ ps (rms).

### Measurement for a few-ps bunch

**Figure 10:**
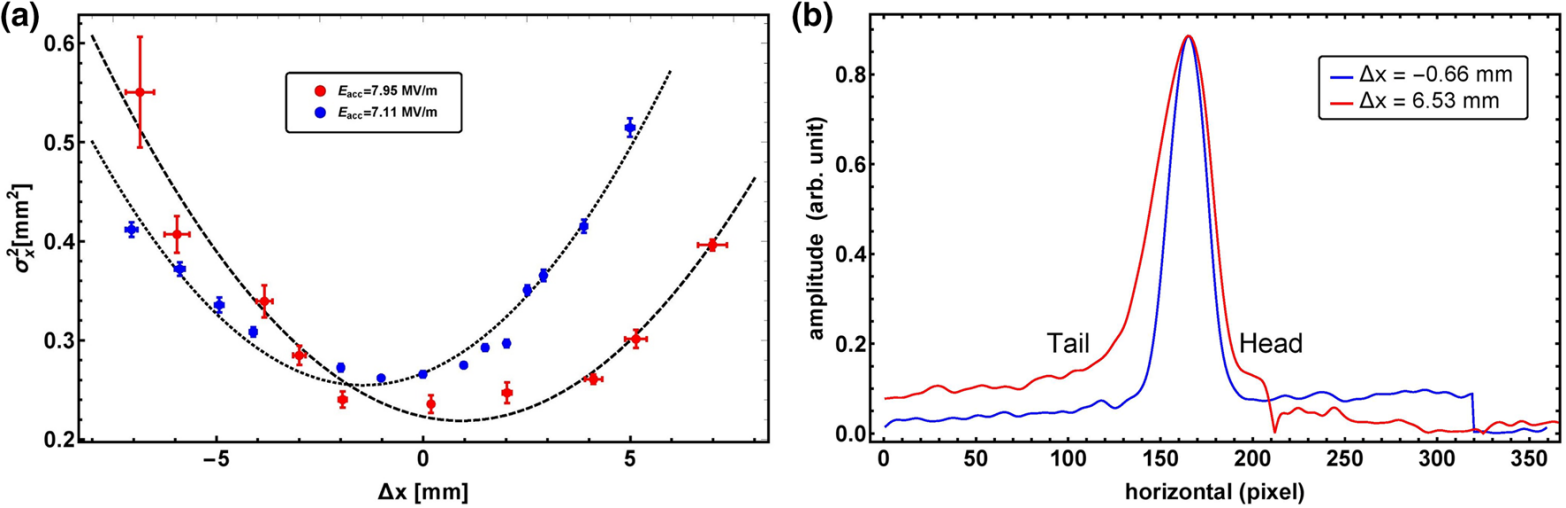
Result of the measurements of (**a**) the relation between the horizontal beam offset and beam size at the profile monitor with peak on-axis accelerating fields of 7.11 and 7.95 MV/m, and (**b**) temporal profile with beam offsets of − 0.66 mm and 6.53 mm. This shows a long tail produced by the GaAs cathode.

In the previous section, the principle of our method was confirmed by measuring the temporal profile for a the long bunch length. It is necessary to verify the accuracy of our method for few-ps bunch lengths. We then measured the bunch length for a single Gaussian pulse whose bunch length is 3.3 ps (rms). Measurements have been performed at the peak on-axis accelerating field $E_{acc}$ of 7.95 MV/m ($\Delta E = 1.62 MeV$) and 7.11 MV/m ($\Delta E = 1.45 MeV$) since the peak on-axis accelerating field strength influences on the accuracy of the bunch length measurement. The beam offset inside the accelerating RF cavity should exceed 4.80 mm to measure a bunch length of 3.3 ps (rms). The results are presented in Fig. 10.

Using the measured result, the value of $\xi$ was estimated as $0.00488 \pm 0.000471$ for $E_{acc}$ = 7.95 MV/m and $3.39 \pm 0.12$ ps (rms) for $E_{acc}$ = 7.11 MV/m. The bunch length was calculated to be $2.94 \pm 0.15$ ps (rms) for $E_{acc}$ = 7.95 MV/m and $0.00558 \pm 0.000389$ for $E_{acc}$ = 7.11 MV/m. This result is consistent with expectations because the duration of the laser pulse on the photo cathode is 3.3 ps (rms)^65^. The result of the bunch length measurement with a lower accelerating gradient also agrees with the duration of the laser pulse on the photo cathode. Thus, the results demonstrate that the proposed method is suitable for few-ps bunch lengths. Since the beam angle is varied up to 8.5 mrad using the corrector magnet to achieve a beam offset of 6 mm at the center of the cavity, it causes variations of the beam trajectory at the entrance and exit of the cavity. This effect leads to a shift in the minimum beam size position and an increase in the negative horizontal deviation of the measured points from the fitted curve for large $\sigma ^2_x$.

In conclusion, we have proposed and demonstrated an experimental method for measuring few-ps level bunch lengths using the deflecting force of an accelerating RF cavity, whose main function is to increase the beam energy. The ability to perform few-ps level bunch length measurements was validated experimentally using a 2-cell 1.3 GHz SRF cavity in the cERL injector at KEK. Bunch lengths of 3.3 ps (rms) with a bunch charge of 10 fC were measured accurately using the cavity, under the influence of an acceleration field of 7.21 MV/m. The measured values agree strongly with the rms duration of the laser pulse on the photo cathode. The proposed method can be applied to the normal-conducting injectors of the many accelerators worldwide which have an S-band cavity with low beam energy because the S-band cavities yield a higher gradient. The most critical aspect towards a sub-ps resolution at high bunch charge involves the nonlinear effects of wakefield and space-charge. It is necessary to estimate carefully the contribution of these effects on the transverse beam profile^66^.

## Supplementary information

Supplementary information.
