# Supplementary material for: Measurement of bunch length and temporal distribution using accelerating radio frequency cavity in low-emittance injector
Source: Sci Rep. 2020 Nov 3;10:18905. doi: 10.1038/s41598-020-76054-w (PMC7609657; doi:10.1038/s41598-020-76054-w)
Supplement: Supplementary file 1 — Supplementary information. [file 41598_2020_76054_MOESM1_ESM.pdf]

Measurement of Electron-beam Bunch Length and Temporal Distribution Using Accelerating Radio Frequency Cavity in High-Brightness Injectors: supplemental document

JI-GWANG HWANG[1], **TSUKASA MIYAJIMA**[2,*], **YOSUKE HONDA**[2], **AND EUN-SAN KIM**[3,†]

[1] *Helmholtz-Zentrum Berlin (HZB), Albert-Einstein straβe 15, Berlin, 12489, Germany.*
[2] *KEK, High Energy Accelerator Research Organization, 1-1 Oho, Tsukuba, Ibaraki 305-0801, Japan.*
[3] *Department of Accelerator Science, Korea University Sejong Campus, Sejong 339-700, South Korea.*
[*] *Corresponding author: tsukasa@post.kek.jp*
[†] *Corresponding author: eskim1@korea.ac.kr*

A. TRANSVERSE MOTION WITH ON-CREST PHASE

For low energy beams, the 6-D particle motion is simulated using the GPT code to analyze the transverse motion with the on-crest phase and $\Delta t = 0$. At $t = 0$, an electron whose initial kinetic energy T_0 is starts from $z = 0$ with the initial coordinates $x_0 = 1$ mm, $x'_0 = 0$ mrad, $y_0 = 0$ mm, and $y'_0 = 0$ mrad. The electron trajectories calculated for various initial kinetic energies are shown in Fig. S1. The upper graph of Fig. S1 shows that the low energy electron is strongly affected by the fringe field around the entrance of the cavity, $z = 0.4$ m. The final positions x_1 as a function of T_0 are shown in Fig. S2. The transverse motion for low energy beams is complicated owing to the fringe field and velocity changes inside the cavity. As T_0 increases from 200 keV to 700 keV, x_1 decreases reaches its minimum value at T_0 = 700 keV, and then increases as T_0 increases.

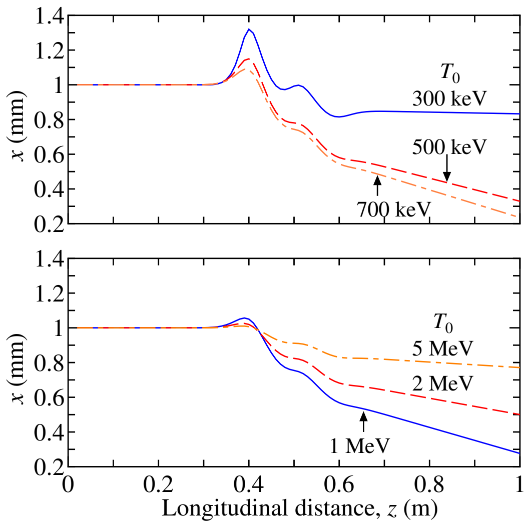

Fig. S1. Electron trajectories corresponding to different initial kinetic energies. The upper graph shows the trajectories when $T0$ = 300 keV, 500 keV and 700 keV. The lower graph shows the trajectories when T_0 = 1 MeV, 2 MeV and 5 MeV. The RF phases are adjusted to the on-crest phases, $\phi_m(T_0)$.

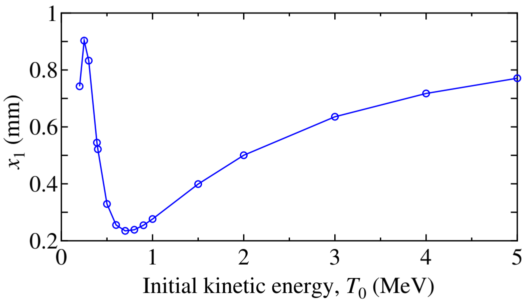

Fig. S2. Electron position at the exit of beamline x_1 as a function of initial kinetic energy T_0.

B. DEFLECTING FORCE IN THE RF CAVITY

We consider the deflecting force experienced by the electron in the cavity when T_0 = 500 keV and 10 MeV. The trajectories are simulated using the initial condition (x_0, x_0') = (1 mm, 0 mrad). The transverse force experienced by the electron is described by the relativistic form of the Lorentz force. The upper graphs in Fig. S3 shows $F_x(z)$ for T_0 = 500 keV and 10 MeV in the left and right column, respectively. The force experienced by the electron for 500 keV depends on $\Delta\phi$. When T_0 = 10 MeV, the dependence of F_x on $\Delta\phi$ is smaller compared to the force for 500 keV.

The integral of F_x is described by

$$\Delta p_x(z) = \int_{\Delta t}^{t(z)} F_x(t')dt', \tag{S1}$$

where $t(z)$ is the arrival time of the electron at z. The quantity $\Delta p_x(z) = p_x(z) - p_x(0)$ is the variation of the horizontal momentum from tis initial value $p_x(0)$. In this case, the initial momentum is zero since $x_0' = 0$. Thus, $\Delta p_x(z)$ is the same as $p_x(z)$. The middle graphs in Fig. S3 show the integral of F_x. The horizontal momentum at the exit of the beamline corresponding to the kinetic energy of 500 keV depends strongly on $\Delta\phi$. When the initial kinetic energy is 10 MeV, the dependence is smaller than that for 500 keV. The horizontal angle of the electron is given by

$$x' = \frac{v_x}{v_z} = \Delta p_x / p_z. \tag{S2}$$

This equation indicates that x' decreases when p_z increases. The lower graphs in Fig. S3 show the results for x'. The x′ at the exit of the beamline corresponding to a kinetic energy of 500 keV depends on $\Delta\phi$. In case of 10 MeV, x' in the cavity is smaller than that for 500 keV, and its dependence on $\Delta\phi$ is smaller than that for 500 keV. The above results lead us to conclude that the effect of $\Delta\phi$ on x' is lower for higher initial kinetic energies.

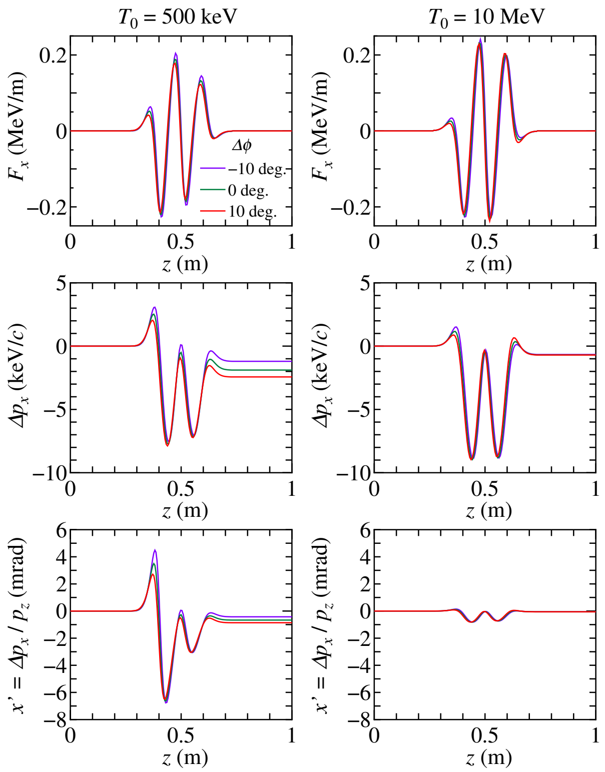

Fig. S3. Deflecting force experienced by the electron with various time differences (upper graphs), its integral (middle graphs), and $x' = \Delta p_x / p_z$ (lower graphs). The left and right columns show the results corresponding to an initial kinetic energy of $T_0 = 500 keV$ and 10 MeV, respectively. The initial condition is x_0 = 1 mm and x'_0 = 0 mrad.
